# The missing element in urology training: operative dictation skills

**DOI:** 10.1186/s12909-025-07032-x

**Published:** 2025-03-30

**Authors:** Coşkun Kaya, Mehmet Erhan Aydin, Cem Tuğrul Gezmiş, Arif Kalkanli, Mazhar Ortaç, Ateş Kadioğlu

**Affiliations:** 1 Department of Urology, University of Health Sciences Eskişehir City Health Application and Research Center, Eskişehir, Turkey; 2 Department of Urology, Taksim Education and Research Hospital, İstanbul, Turkey; 3 Department Urology, Medical Park Gebze Hospital, Kocaeli, Türkiye; 4 Department of Urology, Istanbul University Istanbul Medical Faculty, İstanbul, Turkey

## Abstract

**Aim:**

Operative dictation (OD) is a critical component of surgical documentation, yet its formal teaching is often overlooked in residency training. This study aimed to evaluate the inclusion of OD training in urology residency programs and assess awareness and practices among urology residents (URs) and staff urologists (SUs).

**Materials and methods:**

A structured questionnaire was developed to explore OD practices, awareness, and training methods. The questionnaire consisted of four sections: demographic characteristics, OD practices, awareness of OD as a skill, and formal OD training. It was distributed via e-mail to URs and SUs whose contact information was obtained from the Turkish Urological Association’s Central Anatolia Branch. Descriptive statistics were used to analyze the data.

**Results:**

The survey was sent to 252 participants and achieved a response rate of 86.9% (*n* = 219). Among the respondents, 61 were URs (27.8%) and 158 were SUs (72.1%). Most participants (96.7% of URs and 98.2% of SUs) reported no formal OD training during residency, informal methods, such as reviewing old ODs and guidance from senior residents, were commonly cited as learning approaches. Despite recognizing the importance of structured OD templates, participants highlighted the absence of standardized training curricula. Similar responses from experienced and novice surgeons suggest that this gap has persisted for decades.

**Conclusion:**

This study highlights the lack of formal OD training in urology residency programs and the reliance on informal methods. Integrating standardized OD training modules into residency curricula is essential to improve documentation quality and medico-legal reliability. Future research should explore the effectiveness of educational interventions and develop universal guidelines for OD practices.

**Clinical trial number:**

Not applicable.

**Supplementary Information:**

The online version contains supplementary material available at 10.1186/s12909-025-07032-x.

## Introduction

Operative dictation (OD) is an essential component of surgical documentation that ensures the accurate recording of operative details for future reference. High-quality OD is critical for managing perioperative complications, planning subsequent procedures, and addressing medico-legal and billing requirements. Despite its significance, the quality of OD often falls short of established standards. For instance, a recent study found that only 56.1% of laparoscopic appendicectomy notes adhered to recommended guidelines, highlighting the widespread inconsistency in OD practices [1]. Several organizations, such as the Royal College of Surgeons, have published detailed guidelines to standardize OD practices [2, 3]. These efforts have been complemented by the development of scoring systems to assess OD quality [4–8]. Despite these advancements, compliance with such guidelines remains suboptimal, largely due to the fact that, in surgical practice, the ability to compose an effective OD is typically acquired through informal means, such as observing senior colleagues, reviewing previously written operative notes, or drawing on personal experiences [9]. However, these methods are inherently inconsistent and lack standardization. Operative notes are often based on outdated or non-standardized templates, leading to variability in both their structure and content. The absence of formal, structured training programs in OD not only hampers the systematic development of this essential skill but also risks compromising the quality and reliability of surgical documentation. A 2014 systematic review reported that only 12–25% of residency programs worldwide included formal OD training, underscoring a critical gap in surgical education [9].

The aforementioned problems are equally relevant in urology, a subspecialty of surgery, where the quality of ODs is of critical importance. However, the literature contains limited studies emphasizing the significance of high-quality operative notes in urology [10–13]. Moreover, to the best of our knowledge, no studies have examined whether training in writing ODs is included in urology residency programs.

In this study, we sought to investigate, for the first time in the literature, the inclusion of OD training within urology residency programs on a national platform. By highlighting this previously unexplored aspect of surgical education, we aim to contribute to the discourse and promote awareness regarding the necessity of integrating formal OD training into residency curricula.

## Materials and methods

Following ethical approval, a structured questionnaire was developed by the researchers to evaluate the awareness and training methods of OD within urology residency programs. The questionnaire was developed through a multi-step process. First, a comprehensive literature review was conducted to identify key themes related to OD training and surgical documentation practices. Second, a panel consisting of senior urologists, surgical educators, and experts in medical education reviewed the questionnaire draft to evaluate its clarity, relevance, and comprehensiveness. Their feedback led to refinements, ensuring that the final version of the questionnaire addressed core aspects of OD awareness, training experiences, and perceived gaps in education. The final questionnaire was approved by the expert panel before distribution. It consisted of four sections: [1] demographic characteristics of participants, including age, gender, and years of professional experience; [2] information on who dictated operative notes within their clinical settings; [3] awareness of OD as a professional skill; and [4] the presence of education and training on OD during residency (Additional File).

The questionnaire was distributed in survey format via e-mail to urology residents (URs) and staff urologists (SUs). The contact information of the participants was obtained from the database of the Turkish Urological Association’s Central Anatolia Branch. Participants were included in the study if they were currently practicing as URs or SUs in accredited institutions. Two reminder e-mails were sent at one-week intervals to maximize the response rate.

All responses were collected anonymously to ensure confidentiality. Data were analyzed using descriptive statistics to summarize demographic variables and survey responses. Analytical measures included minimum, maximum, frequency, and percentage values to provide a comprehensive overview of the findings. The data analysis was performed using statistical software to ensure accuracy and reliability.

## Results

### Demographics

E-mails containing the questionnaire were sent to 252 recipients, resulting in a total of 219 responses, achieving a response rate of 86.9%. Among the participants, 61 (27.8%) were URs, and 158 (72.1%) were SUs (Table 1). The median age of the participants was 37 years, ranging from 25 to 72. For URs, the median age was 29 years (range: 25–37), while for SUs, it was 40 years (range: 28–72). The majority of respondents were male, 96.8% (*n* = 212), and only 3.2% (*n* = 7) female. Gender distribution showed slightly more diversity among URs, with 91.8% male and 8.2% female, compared to 98.7% male and 1.3% female among SUs. Regarding professional experience, the median years of experience was 7 years (range: 1–49). URs had a median experience of 3 years (range: 1–5), while SUs had a broader range, with a median of 10 years (range: 1–49).

**Table 1:** Demographic data of the participants

	Total (*n*: 219)	Urology Residents (*n*: 61)	Staff Urologists (*n*: 158)
Age (n) (median, min-max)	37 (25–72)	29 (25–37)	40 (28–72)
Gender (n, %)			
Male Female	212 (96,8) 7 (3.2)	56 (91.8) 5 (8.2)	156 (98.7) 2 (1.3)
Years experience (n) (median, min-max)	7 (1–49)	3 (1–5)	10 (1–49)

### OD practices

The responsibility of composing ODs differed between URs and SUs (Table 2). While 37.7% of URs reported writing their own ODs, the majority (62.3%) indicated that this task was performed by someone else. In contrast, 56.3% of SUs wrote ODs themselves, whereas 43.7% delegated this responsibility. A substantial proportion of participants had reviewed their ODs, with 93.4% of URs and 90.5% of SUs reporting such experience. Feedback on the quality of ODs was also commonly received, with 86.9% of URs and 74.1% of SUs indicating they had received feedback. Despite this, only 34.4% of URs and 33.5% of SUs perceived their ODs as requiring improvement. The majority of participants recognized the importance of structured templates for ODs, with 91.8% of URs and 87.3% of SUs supporting their use in surgical practice.

**Table 2:** Responses of the participants to the questions

		Urology Residents (*n*: 61)	Staff Urologists (*n*: 158)
In your clinic who wrote the OD? (n, %)	Me	23 (37.7)	89 (56.3)
	Another person	38 (62.3)	69 (43.7)
Have you ever needed to review your OD for any reason? (n, %)	Yes	57 (93.4)	143 (90.5)
	No	4 (6.6)	15 (9.5)
Have you ever received feedback about your dictations (n, %)	Yes	53 (86.9)	117 (74.1)
	No	8 (13.1)	41 (25.9)
Are your dictations in need of improvement (n, %)	Yes	21 (34.4)	53 (33.5)
	No	40 (65.6)	105 (66.5)
Are structured templated ODs needed in surgical practice? (n, %)	Yes	56 (91.8)	138 (87.3)
	No	5 (8.2)	20 (12.7)
Does, or did, your surgical curriculum have formal teaching methods for OD? (n, %)	Yes	2 (3.3)	3 (1.8)
	No	59 (96.7)	155 (98.2)
If no, where did you learn how to write an OD? (n, %)	Old, reviewed ODs	32 (52.5)	37 (43)
	From the senior residents	48 (78.7)	59 (68.6)
	From the staff urologists at the clinic	6 (9.8)	9 (10.5)
	From the trainers	3 (4.9)	9 (10.5)
	Other sources (Internet etc.)	1 (1.6)	0 (0)

OD: operative dictations

### Training methods

However, formal training on OD writing was reported to be absent in the surgical curricula of most respondents, with only 3.3% of URs and 1.8% of SUs indicating exposure to formal teaching methods. For those lacking formal training, the primary sources of learning were reviewing old ODs (52.5% of URs and 43% of SUs) and receiving guidance from senior residents (78.7% of URs and 68.6% of SUs). A smaller proportion of participants reported learning from SUs (9.8% of URs and 10.5% of SUs) or trainers (4.9% of URs and 10.5% of SUs). Other resources, such as the Internet, were rarely utilized.

## Discussion

Operative dictation serves as a critical medical record that details the specifics of surgical procedures. Fully completed and standardized OD not only provides high-quality data for patient care and clinical review but also plays a vital role in medico-legal documentation and billing processes. Accurate and detailed OD is particularly significant for medico-legal cases, where essential operative findings can influence legal outcomes. Furthermore, OD contributes to surgical research and quality assurance initiatives and serves as an educational tool for surgical residents, offering opportunities for skill development and professional growth [14].

Despite the critical importance of ODs in surgical practice, the overall quality of OD documentation remains suboptimal. Numerous studies across various surgical disciplines have highlighted significant deficiencies, including incomplete, inconsistent, and non-standardized notes [9, 14–17]. These shortcomings not only jeopardize patient safety and the medico-legal reliability of surgical documentation but also hinder effective communication among healthcare professionals.

While the quality of ODs has been extensively studied in general surgery and other disciplines, there is a notable scarcity of research specifically focused on OD practices within urology. Few studies have addressed the quality of urological ODs or explored strategies to enhance their accuracy and standardization [10–13]. Dukic et al. (2010) evaluated handwritten urological operative notes and compared them with computerized notes based on the English Royal College of Surgeons’ guidelines for Good Surgical Practice [13]. Their findings demonstrated that computerized notes achieved superior documentation quality, highlighting the potential of digital tools in improving OD practices. Similarly, an audit of a computer-based nephrectomy OD application showed that this tool facilitated faster dictation and yielded higher completeness rates for operative notes. Another study conducted an audit based on the English Royal College of Surgeons’ guidelines and found that structured feedback and education significantly improved OD quality among urology and surgery residents [11]. More recently, an audit on transurethral resection of bladder tumor operative notes aligned with the European Association of Urology 2022 guidelines identified key steps for bladder tumor resections [12]. This study concluded that implementing educational programs could enhance the completeness and technical accuracy of TURBT notes. These findings underscore the need for further research and structured interventions to improve OD practices in urology, a field where precise documentation is crucial for both clinical outcomes and medico-legal reliability. In addition, Operative Dictations in Urologic Surgery was published in the United States of America by Wiley in 2019 [18]. The authors of this publication reviewed operative reports for medico-legal issues and identified frequent inadequacies and deficiencies in detailing surgical procedures. To address these gaps, the book provides dedicated OD texts for over 120 of the most common surgical procedures. Each procedure is described in a stepwise, methodical, and detailed format, offering a valuable resource for improving the quality and standardization of urological operative notes.

Globally, the importance of teaching OD writing skills during surgical training has not been sufficiently emphasized. A 2014 systematic review, which included 13 survey-based studies, reported that only 12–25% of residency programs incorporated formal teaching programs on OD [9]. This significant gap in surgical education highlights a lack of structured initiatives to ensure high-quality operative documentation. In recent years, the literature has increasingly emphasized the need for audits to enhance OD quality at both resident and senior surgeon levels. Residency programs have begun incorporating audits as a tool to teach residents how to dictate operation notes effectively and improve their overall quality [19]. These audits not only serve as a feedback mechanism but also provide an opportunity to standardize OD practices and address deficiencies in documentation. By systematically evaluating operative notes, these efforts aim to instill the importance of accuracy, completeness, and standardization in surgical documentation, benefiting both patient care and medico-legal processes. Despite these efforts, literature suggests that OD training remains an underdeveloped aspect of surgical education worldwide, with no universally accepted curriculum or assessment framework.

While the literature in other surgical fields has increasingly focused on audits and formal training to improve OD quality, there are no studies specifically examining whether OD writing skills are formally taught during urology residency. This lack of research in the field of urology further underscores the systemic neglect of this critical aspect of surgical education. Residency programs in general have started incorporating audits to teach residents how to dictate operation notes effectively and improve their quality [20]. These audits, combined with structured feedback, have been shown to enhance the accuracy, completeness, and standardization of operative notes, yet their implementation within urology remains largely unexplored.

This study highlights significant gaps in the formal training of OD skills within urology residency programs. Despite the critical role of ODs in surgical documentation, the majority of participants, including both URs and SUs, reported an absence of structured education on OD writing during their training. Instead, they predominantly relied on informal learning methods, such as reviewing old ODs and seeking guidance from senior residents. Notably, the similarity in responses between participants with extensive professional experience and those early in their careers suggests that this educational gap has persisted for decades, reflecting a systemic issue in surgical education. Furthermore, while most respondents acknowledged the importance of structured OD templates and recognized the need for improvement, the lack of formal training curricula underscores the neglect of this critical skill in urology education. By addressing these gaps and incorporating comprehensive OD training modules into residency programs, it is possible to enhance the accuracy, standardization, and medico-legal reliability of operative notes, ultimately improving the quality of surgical documentation and patient care.

This study has several strengths that enhance its contribution to the literature. First, it is the first study to evaluate the inclusion of OD training specifically within urology residency programs, addressing a critical gap in surgical education research. Second, the study’s national scope provides a comprehensive overview of OD practices and awareness among URs and SUs, making the findings broadly applicable to similar educational contexts. Third, the high response rate (86.9%) ensures the reliability of the data and minimizes the risk of selection bias. Additionally, the structured questionnaire used in this study was developed based on a thorough literature review and expert consultation, ensuring the relevance and clarity of the questions. Finally, by identifying the reliance on informal learning methods and the absence of structured training, this study highlights actionable opportunities for integrating formal OD training into residency curricula, offering a roadmap for improving the quality of surgical documentation in urology.

This study has several limitations that should be considered when interpreting its findings. First, the data were collected through a self-reported survey, which may be subject to response bias as participants might have over- or under-reported their experiences with OD training. Second, the study was limited to participants whose contact information was available in the database of the Turkish Urological Association’s Central Anatolia Branch, potentially excluding other URs and SUs who could have provided additional perspectives. While this sample provided meaningful insights into OD training practices within a defined professional network, it is possible that regional differences in residency curricula or institutional practices exist. This study relied on descriptive statistics to provide a general perspective on OD training trends over time. While comparative statistical analyses could have identified significant differences between subgroups, our primary aim was to highlight the overall lack of structured OD training rather than to assess subgroup variations. Lastly, while the survey evaluated awareness and practices related to OD, it did not include an objective assessment of the actual quality of operative notes, which would provide a more comprehensive understanding of the effectiveness of current training methods.

Future studies should focus on developing and evaluating structured OD training modules tailored specifically for URs. These modules could incorporate interactive workshops, standardized templates, and regular audits to enhance the accuracy, completeness, and medico-legal reliability of operative notes. Additionally, longitudinal studies are needed to assess the long-term impact of such training on the quality of operative documentation and patient outcomes. Further research should also include objective assessments of OD quality across various surgical disciplines, providing comparative insights that can inform best practices. Exploring the integration of digital tools and artificial intelligence to support OD writing represents another promising avenue for improving efficiency and standardization. The reliance on informal learning methods may be a common issue in surgical education worldwide. Future studies should investigate OD training practices in different countries to determine whether similar deficiencies exist in other residency programs. Such studies could provide comparative insights into best practices and contribute to the development of internationally standardized OD training modules. Future studies should incorporate standardized scoring systems, to evaluate the completeness, accuracy, and standardization of OD samples. Such analyses would provide a more comprehensive understanding of the effectiveness of current training methods and the impact of structured OD education. Future studies should aim to include a broader sample encompassing urologists from different regions and institutions to improve the generalizability of findings and provide a more comprehensive assessment of OD training in urology.

Finally, collaboration between urology education stakeholders, including residency program directors and professional associations, is essential to establish universal guidelines and incorporate OD training into surgical curricula on a broader scale. To effectively integrate OD training into residency programs, several strategies can be considered. First, OD education can be incorporated into existing surgical training modules, ensuring that residents receive structured instruction alongside their operative experience. Second, the development and implementation of standardized OD templates can provide a consistent framework for documentation. Third, residency programs can adopt regular OD audits with structured feedback sessions, allowing trainees to refine their dictation skills under the guidance of experienced surgeons. Additionally, digital tools, including AI-assisted OD generators and electronic medical record-integrated dictation systems, can be explored to enhance efficiency and standardization. By adopting these strategies, OD training can be systematically integrated into urology residency programs, improving documentation quality, medico-legal reliability, and overall surgical education.

## Conclusion

This study underscores the critical gaps in OD training within urology residency programs and highlights the reliance on informal learning methods due to the absence of structured education. These findings emphasize the need for integrating standardized OD training modules into surgical curricula to improve the quality, accuracy, and medico-legal reliability of operative notes. Addressing these deficiencies through targeted educational reforms will not only enhance surgical documentation but also contribute to better patient care and professional development for future urologists.

## Electronic supplementary material

Below is the link to the electronic supplementary material.

Supplementary Material 1

## Data Availability

The datasets used and/or analysed during the current study are available from the corresponding author on reasonable request.

## Abbreviations

OD: Operative Dictation
SUs: Staff Urologists
URs: Urology Residents

## References

[1] Shah S, Refalo A, Ebied H, Hammad A. An audit to identify adherence of laparoscopic appendicectomy operation notes to Royal college of surgeons good surgical practice standards. Ir J Med Sci. 2022;191(2):825–30. 33715070 10.1007/s11845-021-02567-6

[2] The Royal College of Surgeons of England, Good Surgical Practice. 2014 [Available from: https://www.rcseng.ac.uk/library-and-publications/rcs-publications/docs/good-surgical-practice/

[3] The Royal College of Surgeons of England. Good Surgical Practice 2022 [Available from: https://www.rcseng.ac.uk/standards-and-research/gsp/

[4] Vergis A, Gillman L, Minor S, Taylor M, Park J. Structured assessment format for evaluating operative reports in general surgery. Am J Surg. 2008;195(1):24–9. 18082539 10.1016/j.amjsurg.2007.08.053

[5] Smyth NA, Sabeh KG, Meiyappan K, Conway SA, Dodds SD. Operative reports in orthopaedic surgery: the need for assessment and education. J Surg Educ. 2020;77(1):229–34. 31501066 10.1016/j.jsurg.2019.08.013

[6] Dexter SC, Hayashi D, Tysome JR. The ankle score: an audit of otolaryngology emergency clinic record keeping. Ann R Coll Surg Engl. 2008;90(3):231–4. 18430339 10.1308/003588408X261537 PMC2430432

[7] Tuffaha H, Amer T, Jayia P, Bicknell C, Rajaretnam N, Ziprin P. The STAR score: a method for auditing clinical records. Ann R Coll Surg Engl. 2012;94(4):235–9. 22613300 10.1308/003588412X13171221499865 PMC3957501

[8] Ho MY, Anderson AR, Nijjar A, Thomas C, Goenka A, Hossain J, et al. Use of the CRABEL score for improving surgical case-note quality. Ann R Coll Surg Engl. 2005;87(6):454–7. 16263017 10.1308/003588405X60687 PMC1964114

[9] Dumitra S, Wong SM, Meterissian S, Featherstone R, Barkun J, Fata P. The operative dictation: a review of how this skill is taught and assessed in surgical residency programs. J Surg Educ. 2015;72(2):321–9. 25456406 10.1016/j.jsurg.2014.09.014

[10] Hoffer DN, Finelli A, Chow R, Liu J, Truong T, Lane K, et al. Structured electronic operative reporting: comparison with dictation in kidney cancer surgery. Int J Med Inf. 2012;81(3):182–91. 10.1016/j.ijmedinf.2011.11.008 22217801

[11] Nzenza TC, Manning T, Ngweso S, Perera M, Sengupta S, Bolton D, et al. Quality of handwritten surgical operative notes from surgical trainees: a noteworthy issue. ANZ J Surg. 2019;89(3):176–9. 29148172 10.1111/ans.14239

[12] Guerero DN, Bruce A, Vayalapra S, Menon V, El Hadi M, Khashaba S. Improving the quality of transurethral resection of bladder tumour (TURBT) operative notes following the European association of urology guidelines: A completed audit loop study. Cureus. 2022;14(10):e30131. 36246089 10.7759/cureus.30131 PMC9550198

[13] Dukic I, Shaw G, Dukic M, Hussain M, Al-Buheissi S, Maraj B. A comparison of handwritten to computerised urological operative notes. Br J Med Surg Urol. 2010;3(1):22–4.

[14] Borchert D, Harshen R, Kemps M, Lavelle M. Operative notes teaching:Re-discovery of an effective teaching tool in surgical training. IJS. 2006;8.

[15] Kanters AE, Vu JV, Schuman AD, Van Wieren I, Duby A, Hardiman KM, et al. Completeness of operative reports for rectal cancer surgery. Am J Surg. 2020;220(1):165–9. 31630821 10.1016/j.amjsurg.2019.09.036 PMC7101251

[16] Liu C, Cheifetz R, Brown C, Nichol A, Speers C, Lohrisch C, et al. Do surgeons convey all the details? A provincial assessment of operative reporting for breast cancer. Am J Surg. 2020;219(5):780–4. 32145920 10.1016/j.amjsurg.2020.02.053

[17] Eichholz AC, Van Voorhis BJ, Sorosky JI, Smith BJ, Sood AK. Operative note dictation: should it be taught routinely in residency programs? Obstet Gynecol. 2004;103(2):342–6. 14754706 10.1097/01.AOG.0000109521.44989.53

[18] Armenakas NA, Fracchia JA, Golan R. Operative dictations in urologic surgery. Wiley; 2019.

[19] Abbas SH, Singh S, Sundran R, Akbari K, Gilmour J, Puttick M. A thorough note: does a procedure-specific operation note proforma for laparoscopic appendicectomy improve compliance with the Royal college of surgeons of England guidelines?? Int J Surg Open. 2016;2:1–5.

[20] Bozbiyik O, Makay O, Ozdemir M, Goktepe B, Ersin S. Improving the quality of operation notes: effect of using Proforma, audit and education sessions. Asian J Surg. 2020;43(7):755–8. 31653554 10.1016/j.asjsur.2019.10.002

