# Supplementary material for: The missing element in urology training: operative dictation skills
Source: BMC Med Educ. 2025 Mar 30;25:461. doi: 10.1186/s12909-025-07032-x (PMC11956247; doi:10.1186/s12909-025-07032-x)
Supplement: Supplementary file 1 — Supplementary Material 1 [file 12909_2025_7032_MOESM1_ESM.docx]

Questionnaire Form

I have read the information letter and by completing this online survey, I agreed to participate in this study

Yes No

What is your age?

Your response: ____ years

What is your gender?

Female Male

What are your years experience?

Your response: ____ years

In your clinic who wrote the operation dictation?

Me Another person

Have you ever needed to review your operation dictation for any reason?

Yes No

Have you ever received feedback about your dictations

Yes No

Are your dictations in need of improvement

Yes No

Are structured templated operation dictations needed in surgical practice?

Yes No

Does, or did, your surgical curriculum have formal teaching methods for operation dictation?

Yes No

If no, where did you learn how to write an operation dictation?

Old, reviewed operation dictations

From the senior residents

From the staff urologists at the clinic

From the trainers

Other sources (Internet etc.)
